# E-Waste Acrylonitrile–Butadiene–Styrene (ABS) Upcycling via Low-Dose Electron Beam Irradiation (EBI) and Halloysite Nanotubes (HNTs): A Circular Approach

**DOI:** 10.3390/ma19183872

**Published:** 2026-09-11

**Authors:** Sofia Fares, Mustapha Kaci, Nadjet Dehouche, Christelle Delaite, Amira Zaouak, José-Marie Lopez-Cuesta

**Affiliations:** 1 Laboratoire des Matériaux Polymères Avancés (LMPA), Faculté de Technologie, Université de Bejaia, Bejaia 06000, Algeria; sofia.fares@univ-bejaia.dz (S.F.); mustapha.kaci@univ-bejaia.dz (M.K.); nadjet.dehouche@univ-bejaia.dz (N.D.); 2 Laboratoire de Photochimie et d'Ingéniérie Macromoléculaire (LPIM), Institut de Recherche Jean-Baptiste Donnet, Université de Haute-Alsace, EA 4567, F-68100 Mulhouse, France; christelle.delaite@uha.fr; 3 Laboratory on Energy and Matter for Nuclear Sciences Development, National Center for Nuclear Sciences and Technologies, Sidi Thabet Technopark, Ariana 2020, Tunisia; amirazaouak@gmail.com; 4 Polymers Composites and Hybrids (PCH), IMT Mines Ales, F-30100 Ales, France

**Keywords:** e-waste ABS, halloysite nanotubes, composites, upcycling, e-beam irradiation

## Abstract

**Highlights:**

- An eco-friendly method combining EBI and HNT reinforcement for e-waste ABS property enhancement.
- At 10 kGy, EBI boosts tensile strength (+9%) and Young’s modulus (+40%) of e-waste ABS.
- With 5 wt% HNTs, EBI lowers optimal crosslinking dose to 5 kGy.
- HNTs + controlled EBI enable valorization into high-performance materials.

**What are the main findings?**

- EBI at 10 kGy optimally enhances unfilled e-waste ABS: tensile strength increases by 9%, Young’s modulus by 40%, and thermal stability (T_5%_) increases by 25 °C, due to radiation-induced crosslinking.
- HNTs (5 wt%) shift the radiation response of the composite: the optimal EBI dose drops from 10 kGy to 5 kGy, where the composite reaches its highest storage modulus (G’) and best thermal stability, indicating a polymer–filler crosslinking effect.
- Dose-dependent competition between crosslinking and chain scission governs all properties of unfilled e-waste ABS: low doses (5 kGy) favor chain scission (lower T_g_, T_5%_, and viscosity) and intermediate doses favor crosslinking (improved mechanical/thermal properties), while higher doses (15 kGy) bring properties back toward the baseline due to renewed degradation confirmed across FTIR, TGA, DSC, tensile, and rheological measurements.

**What are the implications of the main findings?**

- A viable, eco-friendly upcycling route for e-waste ABS: combining low-dose EBI (no solvents/chemical initiators) with HNT reinforcement offers a solvent-free, additive-free method to restore and even exceed the mechanical/thermal performance of degraded recycled ABS, supporting circular economy goals.
- Process optimization is dose- and formulation-specific: since the optimal irradiation dose differs for unfilled ABS (10 kGy) versus HNT-reinforced ABS (5 kGy), industrial implementation requires tailoring EBI parameters to the exact composite formulation to avoid over- or under-irradiation and maximize property gains.
- Trade-offs must be managed for multifunctional applications: while EBI/HNTs improve thermal stability and promote char formation and stiffness, care must be taken regarding potential effects on flame-retardant additives and slight tensile property reductions in the composite, meaning that the approach is promising but needs balancing against application and specific performance requirements (e.g., safety standards of electrical and electronic equipment).

**Abstract:**

In this work, a sustainable approach is proposed for upcycling recycled Acrylonitrile–Butadiene–Styrene (ABS) derived from electrical and electronic waste (e-waste) through a combination of electron beam irradiation (EBI) technique and halloysite nanotubes (HNTs) used as reinforcement. The study reveals that the application of EBI at an optimal dose of 10 kGy enhances the tensile properties of unfilled e-waste ABS, achieving an increase of almost 9% in tensile strength (TS) and about 40% in Young’s modulus (E). Incorporating 5 wt% HNTs into e-waste ABS not only improves the thermal stability of the composite material, but also modifies its behavior towards irradiation, shifting the optimal dose to 5 kGy. Another effect of the combination of HNTs and irradiation is noted with regard to the increase in storage modulus (E’). The findings present a promising and eco-friendly route to transform e-waste ABS into a higher-value engineering material.

## 1. Introduction

The transition towards a circular economy has promoted the research work for advanced recycling technologies capable of transforming low-value waste streams into high-performance materials [1]. One of the most urgent targets for such technologies is to deal with e-waste. Indeed, more than 62 million tons of e-waste was generated worldwide in 2022, averaging nearly 7.8 kg per person annually [2]. Among this large quantity of e-waste, nearly 22% is recycled [3]. Hence, e-waste constitutes an important yet underutilized resource within circular economy frameworks. It contains valuable materials, including plastics, which constitute roughly 20 wt% of its composition. Among these plastics, ABS is reported as the dominant thermoplastic in waste electric and electronic equipment (WEEE), particularly in computer equipment [4]. ABS represents almost 30 wt%, while other recoverable types include mainly high-impact polystyrene (HIPS), polycarbonate (PC) and polypropylene (PP) [5]. Owing to its excellent balance of mechanical performance, processability, dimensional stability, electrical insulation and chemical resistance, ABS remains one of the most valuable polymers which can be recovered from electronic waste streams [6]. However, recycling e-waste ABS through conventional melt-processing techniques poses challenges, out of which difficulty arises from the presence of hazardous substances and additives contained in the plastics formulation, such as brominated flame retardants (5–25 wt%) and antimony trioxide [7]. These substances are harmful to both the environment and human health [8]. Moreover, e-waste ABS exhibits significantly degraded mechanical performance and embrittlement [9] in comparison with pristine ABS as a consequence of both the recycling process and the exposure during its service life. Furthermore, during use, ABS is prone to being submitted to heat, UV light, and mechanical load, which may progressively induce thermo-oxidative and photo-oxidative degradation [10,11]. Moreover, during mechanical recycling, the temperature rise and shear forces applied to e-waste ABS further promote degradation through chain scission [12]. Consequently, recycled e-waste ABS often exhibits inferior mechanical and thermal properties compared to virgin materials, limiting its reuse in demanding applications and, subsequently, reducing its economic value. To overcome these limitations, many research studies, some dating back to the 1990s, have focused on upcycling strategies that not only recycle waste plastics but also restore or enhance their properties [9,13,14,15]. Among the various approaches investigated, the incorporation of nanofillers has emerged as an effective route to improve the performance of recycled polymers [16,17]. Indeed, various nanofillers such as clay-based nanoparticles, carbon nanotubes, metal oxides, and nanocellulose have been successfully used to enhance the mechanical, thermal, rheological and functional properties of recycled polymer composites [18,19]. One of the available nanofillers is HNT, which is a natural, tubular clay that makes an excellent eco-friendly reinforcement. HNTs are cost-effective, thermally stable, and blend well with many polymers [20,21]. Even at low loadings of 1–5 wt%, HNTs have been shown to enhance the mechanical strength, thermal stability, and flame retardancy of recycled plastics when homogeneously dispersed [22]. In a previous work [23], we have demonstrated that adding 5 wt% HNTs to recycled e-waste ABS significantly improves its properties. However, while the reinforcing effect of HNTs is well established in various polymer systems [18,19,20,21,22], its interaction with subsequent irradiation treatment in the specific context of e-waste ABS has not yet been systematically investigated. On the other hand, ionizing radiation offers another innovative and green approach for polymer modification [6,24,25]. For instance, electron beam irradiation (EBI) is a well-established industrial technique that can induce crosslinking, chain scission, and grafting in polymers without requiring chemical initiators or solvents. EBI offers precise control over the modification process through the adjustment of irradiation dose and atmosphere [26]. Consequently, EBI is increasingly viewed not merely as a modification technique but as a powerful tool for radiation-assisted polymer upcycling [27,28]. Previous studies have reported synergistic or modified radiation effects in polymer–nanofiller systems, including silica/graphene-filled natural rubber composites [29,30]. These works indicate that nanofillers may influence the balance between radiation-induced crosslinking and chain scission through interfacial interactions and their effect on radical processes. However, the extent to which such effects occur in e-waste ABS containing HNTs, and, particularly, whether HNTs can shift the optimal irradiation dose, remains unexplored and cannot be inferred solely from prior reports.

In this context, the present study does not report a fundamentally new nanofiller-reinforcement or radiation-modification phenomenon. Instead, our objective is to examine whether HNT incorporation modifies the irradiation response of e-waste ABS and, if so, to determine whether this modification leads to a change in the dose required to achieve the most favorable balance of properties. The possible contribution of HNTs to such dose-dependent behavior is discussed cautiously, based on the evolution of structural, thermal, mechanical, and rheological properties obtained in this work, while direct mechanistic evidence (e.g., radical scavenging or interfacial stabilization) is not yet demonstrated and remains a subject for future investigation.

Therefore, in this work, the potential of low-dose electron beam irradiation, i.e., 5, 10 and 15 kGy, is investigated as an upcycling strategy for recycled WEEE-derived ABS and its halloysite nanotube-reinforced composite containing 5 wt% HNTs. Particular attention is devoted to understanding the effects arising from the combination of nanoreinforcement and irradiation treatment, especially on thermal and mechanical properties. The effect of both EBI and nanoreinforcement on the structural, thermal, tensile, and rheological properties of e-waste ABS has been investigated by several techniques, including ATR-FTIR spectroscopy, XRD, TGA/DTG, and DSC, as well as tensile and rheological measurements. Moreover, we aim to identify the optimal irradiation dose that maximizes property improvements for both the recycled matrix and its HNT-based composite, while providing an experimentally grounded discussion of the contribution of HNTs to this irradiation-based upcycling approach.

## 2. Materials and Methods

### 2.1. Materials

ABS was recovered from WEEE collected at a local landfill in Bejaia (Algeria). The feedstock consisted of a single homogenized batch comprising a 50/50 wt% mixture of computer monitor casings and landline telephone frames, all of white color. A pre-sort by color (white only) was deliberately performed to reduce visual and compositional variability. The selected components from the two WEEE sources were homogenized prior to processing. No sorting according to the degree of degradation was performed. Furthermore, because only a single batch was used, batch-to-batch variability was not assessed. To ensure that the feedstock corresponded specifically to ABS, only components explicitly marked with the “ABS” identification symbol were selected and retained for further processing. This criterion provides a baseline level of polymer-type consistency. The resulting material was designated as e-waste ABS. EDX analysis (as in our previous work [23]) showed inorganic additives in the e-waste ABS, including bromine and antimony compounds such as Sb_2_O_3_, typical of flame-retardant systems. Table 1 lists the main elements found.

Halloysite nanotubes (HNTs) were supplied by SOALKA Company (Algerian Company of Kaolin, Guelma, Algeria). Prior to use, the raw HNTs were dried in an oven at 90 °C overnight. The dried clay was then ground into a fine powder using a laboratory ball mill (FAURE Equipment SA) and sieved to obtain particles with an average diameter of 25 µm, including a minor fraction of nanoparticles.

### 2.2. Sample Preparation

**Figure 1:**
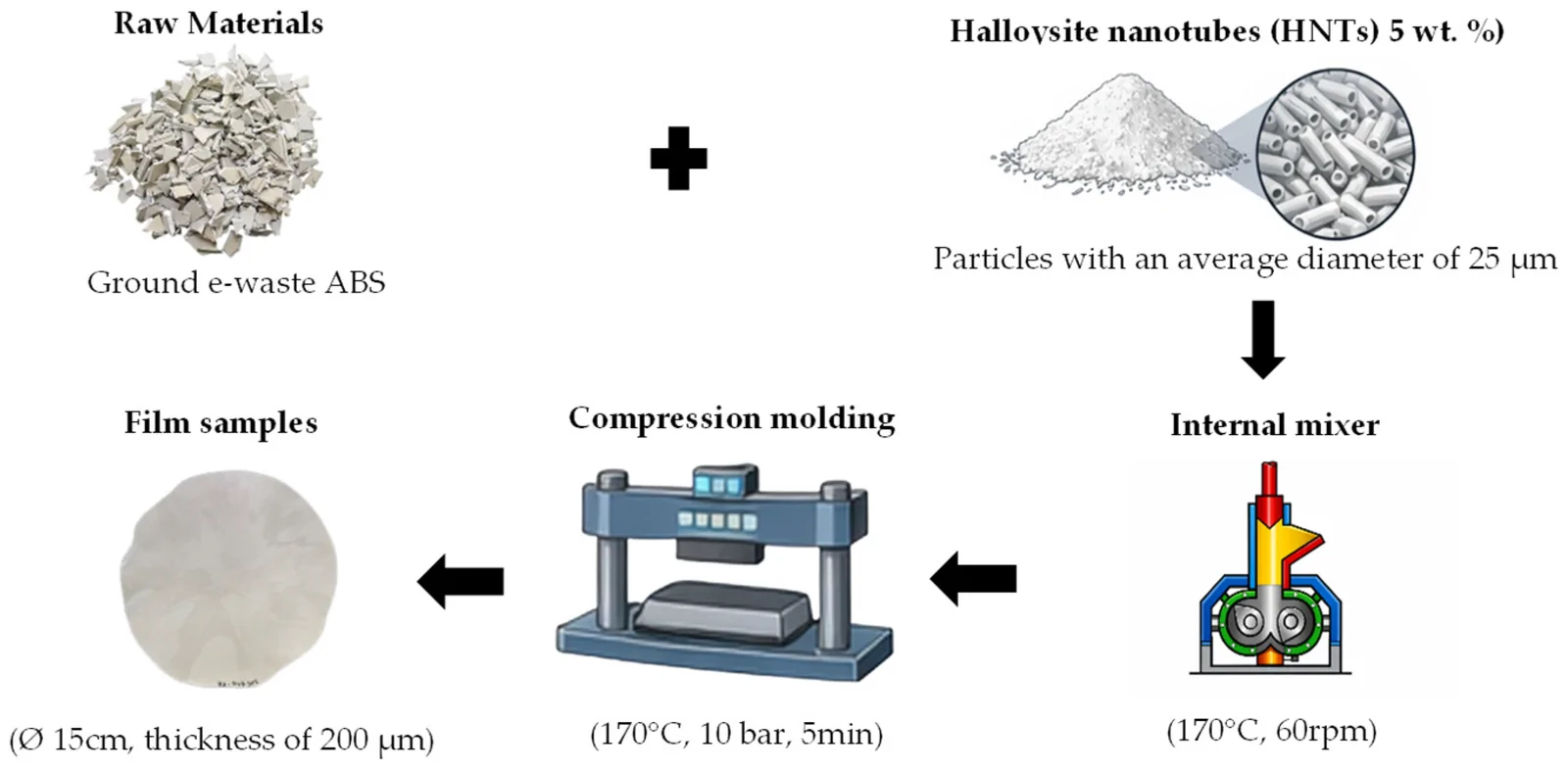
Processing steps for the preparation of e-waste ABS and its HNT-based composite film samples.

Processed HNTs were incorporated into the e-waste ABS matrix at filler content of 5 wt% and blended in a Plasticorder internal mixer (W 50 EHT, New Jersey, USA) at 170 °C, and a screw speed of 60 rpm for 6 min. The resulting compounded materials were ground and pressed into thin films by using a CARVER 3856 CE hydraulic press. The compression molding conditions consisted of a 2 min. preheating phase, followed by 1 min. degassing, and a 2 min. compression stage. All thermal steps were conducted at 170 °C under applied pressure of 10 bar. The samples were cooled under ambient conditions for 10 min. to obtain films with an average thickness of 200 µm. After this, all samples were placed in zip bags away from light and humidity. Processing steps are shown in Figure 1.

### 2.3. E-Beam Irradiation (EBI) Test

Both e-waste ABS and its HNT-reinforced composite were subjected to EBI at the following doses, i.e., 5, 10, and 15 kGy, using a CIRCE III electron accelerator at the Tunisian Center for Nuclear Sciences and Technologies in Sidi Thabet. All irradiation tests were performed in air at ambient temperature, without nitrogen purging or vacuum conditions. EBI process was conducted at three distinct energy levels, 5, 7.5 and 10 MeV, with a maximum power output of 5 kW. To ensure uniform exposure, the samples were carefully placed on trays, and then moved to the irradiation source by a conveyor system. EBI test was performed at constant dose rate of 40 kGy/min. The exposure time was adjusted accordingly to achieve the desired total doses. For accurate dosimetry, a GEX B3 dosimeter was used to monitor and verify the radiation dose received by each sample during the irradiation process.

### 2.4. Characterization Techniques

#### 2.4.1. Attenuated Total Reflectance–Fourier Transform Infrared Spectroscopy (ATR-FTIR)

Changes in chemical structure induced by EBI for both e-waste ABS and e-waste ABS/HNTs (5 wt%) were investigated by ATR-FTIR spectroscopy using a NICOLET IS50 spectrometer (USA) equipped with Diamond/ZnSe crystal. ATR-FTIR Spectra were recorded at room temperature in the range of 4000–650 cm^−1^ by averaging 32 scans at a resolution of 4 cm^−1^.

#### 2.4.2. X-Ray Diffraction Analysis (XRD)

The crystalline structures of both e-waste ABS and HNT-reinforced composite samples were analyzed before and after EBI treatment by X-ray diffraction using a PAN analytical Empyrean multipurpose diffractometer (The Netherlands) with Cu-Kα radiation (λ = 1.5405 Å). Measurements were conducted in reflection mode across a 2θ range of 10–60°, employing a step size of 0.02° and counting time of 30 s/step. The instrument was operated at 45 kV and 40 mA.

#### 2.4.3. Thermogravimetric Analysis (TGA)

Thermal stability of both unirradiated and irradiated e-waste ABS and its HNT-reinforced composite was evaluated by TGA using SETSYS apparatus (Setaram, France). Approximately 10 mg of each sample was heated from 25 to 600 °C at a constant heating rate of 10 °C/min under continuous N_2_ flow. The main parameters, including the onset degradation at 5% weight loss (T_5%_), the temperature at maximum degradation rate (Tmdr), and the percentage of residue at 600 °C, were determined from TGA thermograms. The data reported represent the average of three trials.

#### 2.4.4. Differential Scanning Calorimetry (DSC)

Glass transition temperature (T_g_) of both e-waste ABS and e-waste ABS filled with HNTs were determined by DSC using a Q200 DSC calorimeter (TA Instruments, USA) before and after EBI. Samples of approximately 10 mg were encapsulated in non-hermetic aluminum pans and subjected to various heating and cooling cycles as follows: (1): heating from 20 to 200 °C followed by (2): cooling from 200 to 20 °C at constant heating rate of 10 °C/min, and again (3): a second heating scan from 20 to 200 °C and a cooling cycle at the same rate of 10 °C/min. T_g_ values were obtained from the midpoint of the transition in the second heating rate cycle. Three trials were conducted for each formulation sample.

#### 2.4.5. Tensile Tests

Tensile properties of the film samples were assessed via uniaxial tensile tests on a Zwick typ-8306 universal testing machine equipped with 5 kN load cell. Tests were performed at a constant cross-head speed of 1 mm/min under controlled ambient conditions (23 ± 2 °C, 40% RH). From stress–strain curves, Young’s modulus (E), tensile strength at maximum yield (σmax) and elongation at break (εb) were determined. The average values are the product of seven trials.

#### 2.4.6. Rheological Measurements

Dynamic rheological measurements were carried out to assess the changes in rheological properties of the samples using an ARES rheometer (Rheometric Scientific). Measurements were carried out in oscillatory shear mode with parallel plate equipment at a fixed temperature of 200 °C using 0.5% strain and an angular frequency ranging from 0.1 to 100 rad/s. Complex viscosity (η*) and storage modulus (G’) were determined for both unfilled e-waste ABS and its HNT-reinforced e-waste ABS composite.

## 3. Results and Discussion

### 3.1. Attenuated Total Reflectance–Fourier Transform Infrared Spectroscopy (ATR-FTIR)

**Figure 2:**
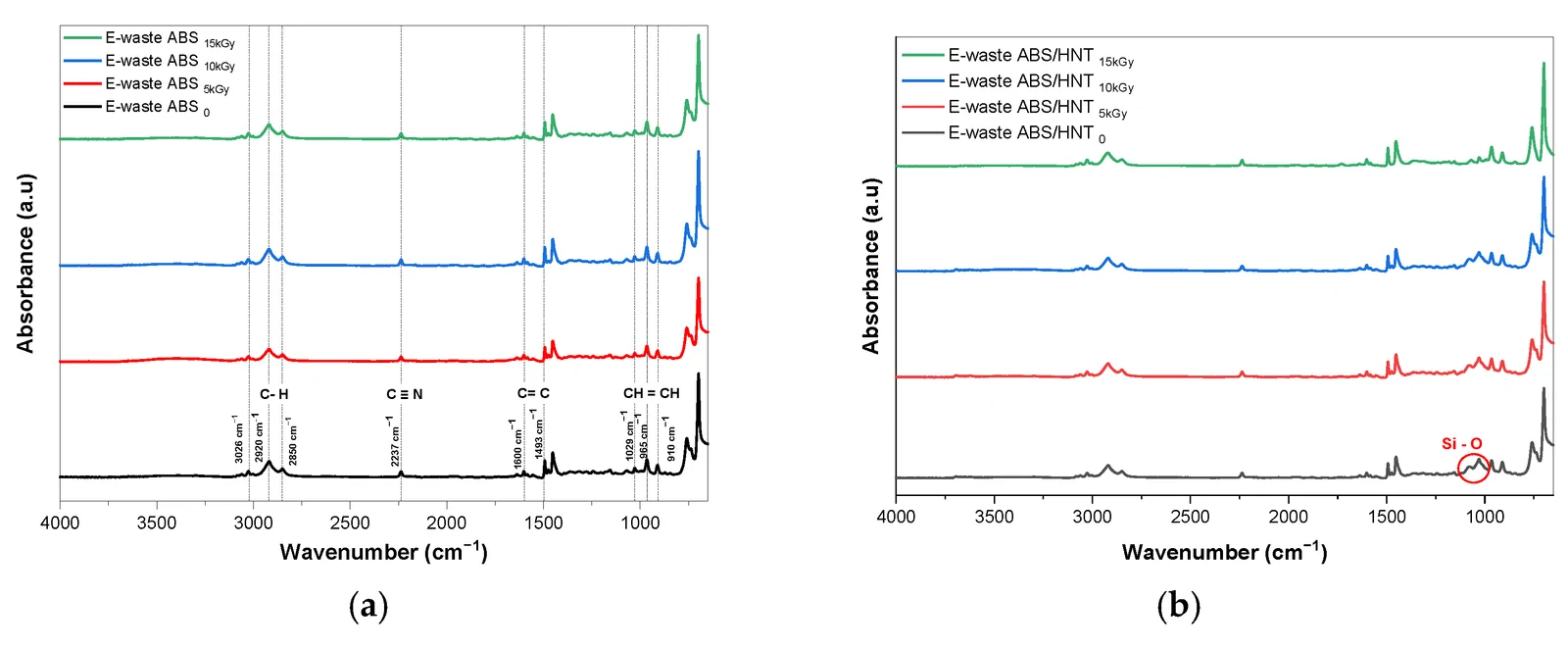
ATR-FTIR spectra of e-waste ABS (**a**) and e-waste ABS/HNTs (5 wt%) (**b**) recorded in the 4000–650 cm^−1^ region before and after irradiation at 5, 10 and 15 kGy.

Changes in the chemical structures of both e-waste ABS and its HNT-reinforced composite (5 wt%) were investigated by ATR-FTIR spectroscopy in the dose range, i.e., 5, 10, and 15 kGy, and the spectra were recorded in the 4000–650 cm^−1^ region. The relative spectra of e-waste ABS and its HNT-reinforced composite are presented in Figure 2a and Figure 2b, respectively. All spectra were normalized to the aromatic ring stretching band at 1602 cm^−1^, which remains almost constant (relative variation below 1%) across the irradiation doses and was therefore used as an internal reference band for quantitative comparison. This normalization approach is formally equivalent to a band ratio analysis (e.g., I_2919_/I_1602_, I_2850_/I_1602_, I_3026_/I_1602_), which is standard practice in ABS degradation studies [31]. This method effectively corrects for local thickness fluctuations and contact pressure variations inherent to ATR-FTIR measurements. From Figure 2a,b, no new absorption bands appear in the FTIR spectra of either e-waste ABS or its HNT-reinforced composite, whatever the dose compared to the unirradiated samples. However, the EBI process induces some intensity changes in many absorption bands such as those located at 2850, 2919, 3026 and 1029 cm^−1^, which vary depending on both the sample type and the applied dose. Regarding the nitrile group (C≡N) at 2237 cm^−1^, its position remains essentially unchanged and its normalized intensity varies by less than 2% across all doses for both sample types, consistent with the higher radiation resistance of the acrylonitrile phase compared to the polybutadiene phase [6]. Similarly, the butadiene unsaturation bands at 965 cm^−1^ and 910 cm^−1^ exhibit only marginal intensity variations (within ±3%) without a clear dose-dependent trend. This is likely due to the already partially saturated state of the polybutadiene phase in the aged e-waste material, combined with the competing effects of crosslinking (consuming double bonds) and chain scission (generating new unsaturations), which may result in the absence of spectral changes at these positions within the relatively low dose range used. To provide a more quantitative assessment of these spectral variations, Table 2 provides the values of the intensity ratios of the main aliphatic and unsaturated C–H bands relative to the internal reference band at 1602 cm^−1^ as a function of irradiation dose. This analysis allows for a direct comparison of the relative evolution of each band and facilitates the identification of dose-dependent trends. Based on the literature data [6,24], the spectral variations suggest the occurrence of chemical transformations in the irradiated materials, which are typically attributed to competing chain scission and crosslinking mechanisms. These phenomena may occur simultaneously in polymeric systems subjected to EBI. Indeed, the literature [32] demonstrated that higher irradiation doses have been reported to promote chain scission, while lower doses promote crosslinking network formation. Some authors [33] argued that the intensity changes in the case of polyether sulfone (PES) subjected to ɣ-irradiation may result from a decrease or increase in the concentration of already existing bonds. This is consistent with our findings where slight differences in FTIR spectra have been observed, but the absorption band positions remain almost unshifted. Only the band intensity of the characteristics groups changes after exposure to EBI. However, it is useful to specify that the intensity variations alone cannot unambiguously distinguish between crosslinking, chain scission, oxidation, or microenvironmental changes (e.g., changes in free volume, molecular packing, or local chain conformation). Thus, the observed trends may result from a combination of these competing phenomena, rather than being attributed to a single mechanism. However, the presence of HNTs in e-waste ABS has an influence on the extent of the spectral changes, due probably to the nanotubes’ effect on the radiation-induced crosslinking efficiency and possibly on the local polymer chain packing and interfacial interactions, which may contribute to microenvironmental variations in the composite. This observation aligns with recent work on e-waste ABS, where the addition of functional additives was shown to modify the polymer’s response to electron beam irradiation, as reported by Zhou et al. [6]. It was argued that EBI chemically alters polymers. For example, in irradiated PEO films, changes in the main FTIR peaks, like a stronger peak at 1100 cm^−1^, indicate that polymer chains are breaking or branching. A growing peak at 1654 cm^−1^ also suggests the occurrence of a new crosslinking fraction [34].

**Figure 3:**
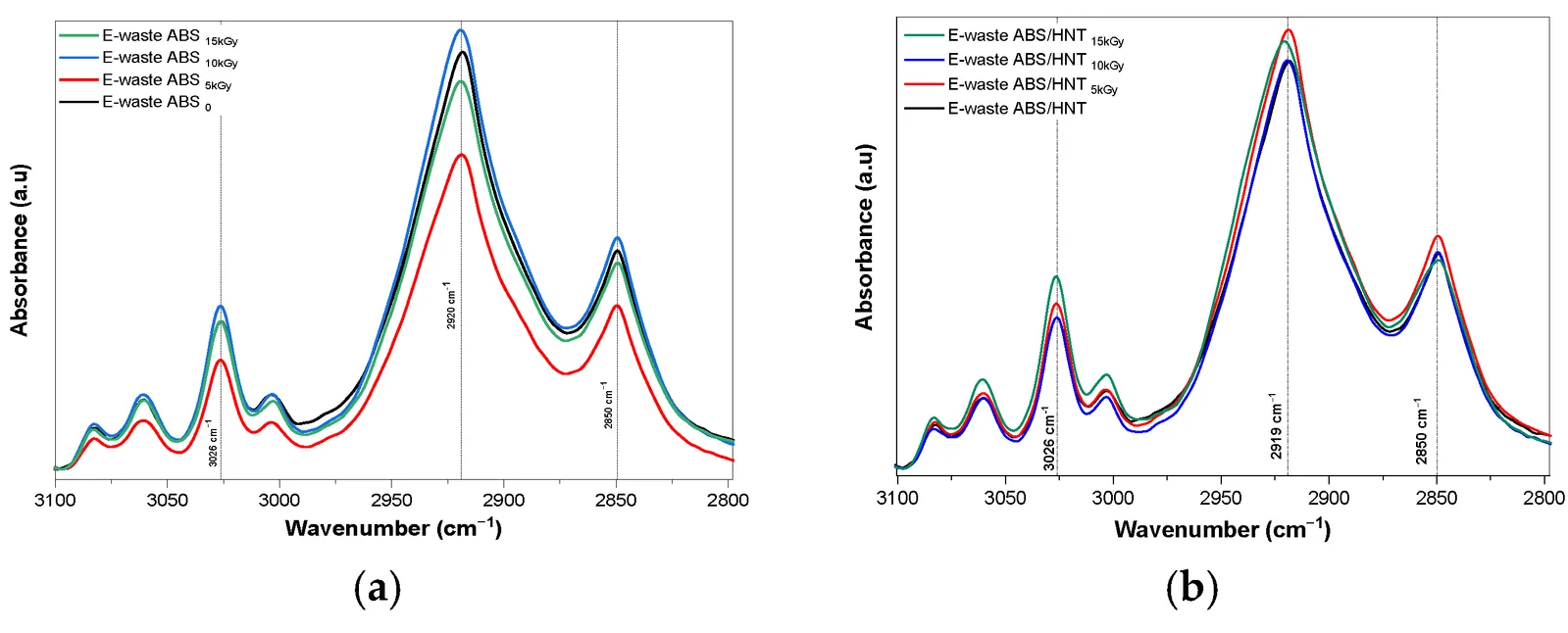
ATR-FTIR spectra of e-waste ABS (**a**) and e-waste ABS/HNTs (5 wt%) (**b**), recorded in the 3100–2800 cm^−1^ region before and after irradiation at 5, 10 and 15 kGy.

Since the FTIR spectra were normalized with respect to the absorption band at 1602 cm^−1^, Figure 3a shows changes in the band intensity at 2850, 2919, and 3026 cm^−1^ for e-waste ABS before and after exposure to EBI. The intensity spectral variations could be attributed to competing effects mainly involving chain scission, crosslinking and oxidation, which are dose-dependent phenomena commonly observed in irradiated polymers.

At the lower dose of 5 kGy, where oxidation processes may dominate, the aliphatic C–H bonds corresponding to the 2850 and 2919 cm^−1^ bands may undergo chain scission (see Table 2). This involves the cleavage of –CH_2_– groups, resulting in a reduction in aliphatic C–H vibrations [35]. Similarly, the aromatic/unsaturated C–H bond at 3026 cm^−1^ may be susceptible to radical attack, potentially inducing ring opening of the styrene component or double-bond cleavage within the butadiene phase. These observations are consistent with studies showing that different phases of the ABS copolymer respond differently to irradiation, with the butadiene phase being particularly susceptible to modification [36]. This selective attack is often attributed to radical species generated in the polybutadiene phase, which can then initiate reactions in the styrene–acrylonitrile matrix [37,38]. In contrast, at doses of 10 and 15 kGy, an increase in the band intensity at 2850 and 2919 cm^−1^ corresponding to aliphatic C–H stretching is observed. This increase is consistent with crosslinking, which enhances the local density of –CH_2_– groups, particularly within the butadiene phase [39]. However, it should be noted that this explanation is not the only one, and the observed increase could also reflect a reduction in chain scission phenomena and also a saturation of oxidative pathways. Nevertheless, the dose-dependent trend observed here aligns with the literature data, which reports that crosslinking becomes increasingly significant at higher doses in ABS systems [32,39]. To further support this interpretation, we examined the band ratio I_2919_/I_1602_, which shows a clear increase at 10 and 15 kGy; however this was more pronounced at 10 kGy, as shown in Table 2. This quantitative trend strengthens the assignment of the intensity increase to crosslinking rather than to random fluctuations or measurement artifacts. The increase may be explained as a result of crosslinking, which enhances the local density of –CH_2_– groups, particularly within the butadiene phase [39]. In Figure 3b corresponding to the HNT-reinforced composite sample, the ATR-FTIR spectral features are less pronounced than in e-waste ABS. However, the composite sample exposed to 5 kGy differs from the others, exhibiting more intense absorption bands, notably at 2919 and 2850 cm^−1^, suggesting that crosslinking may occur at a lower dose in the presence of HNTs. Nevertheless, there are also other alternative explanations, such as improved interfacial adhesion between HNTs and the polymer matrix, or local conformational changes induced by the nanofillers, which may also contribute to the observed spectral enhancement. The presence of HNTs may alter the local refractive index and contact area in ATR measurements, potentially affecting absolute band intensities. However, since all spectra were normalized to the 1602 cm^−1^ band, which remained stable, we consider this effect to be minimal. It is also noted that the presence of additives in e-waste ABS, mainly flame retardants, pigments and others, may influence the material’s radiation response.

**Figure 4:**
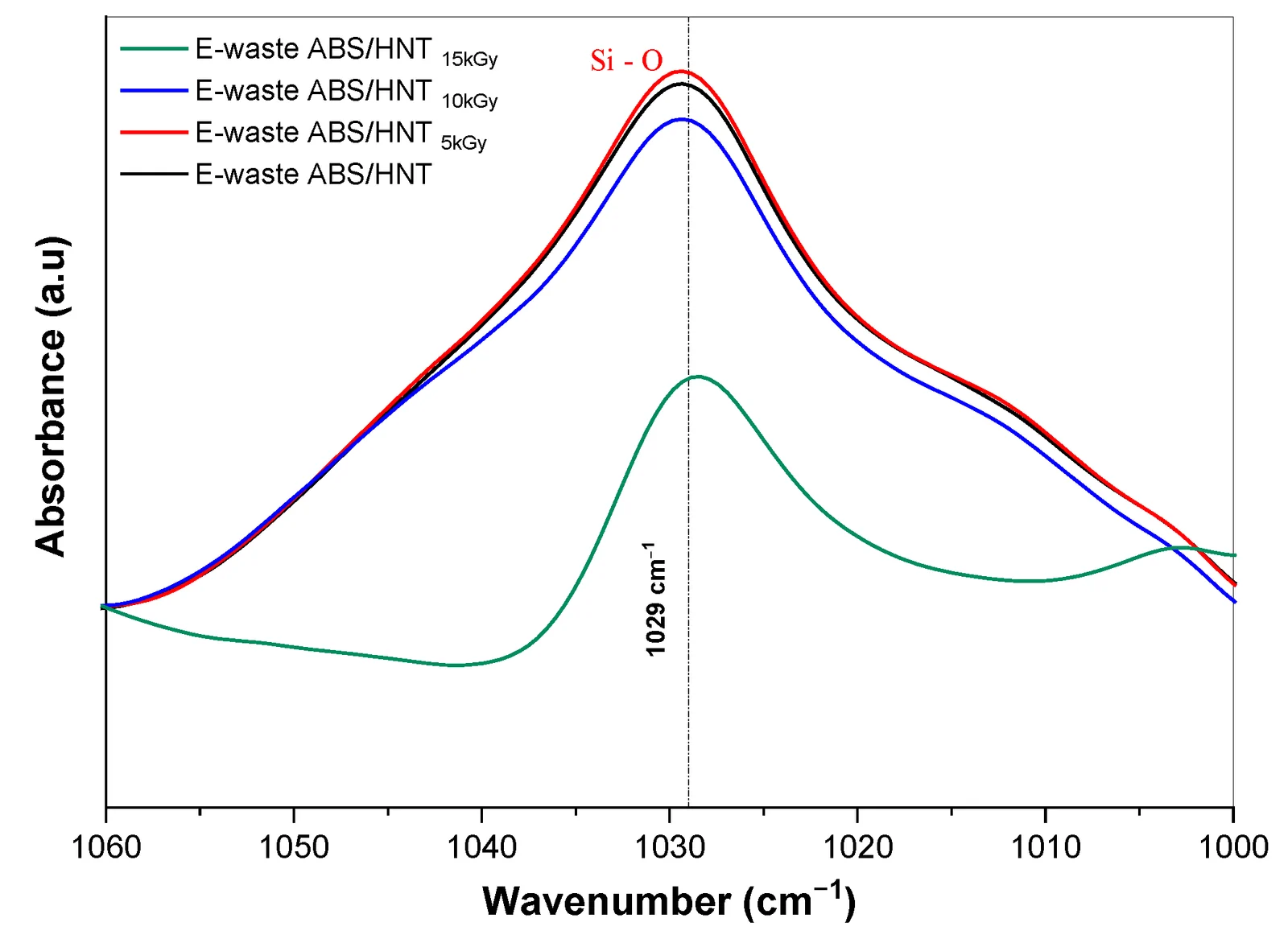
FTIR-ATR spectra of e-waste ABS/HNTs (5 wt%), recorded in the region 1060–1000 cm^−1^ before and after EBI at 5, 10 and 15 kGy.

Previous work has shown that such additives can undergo degradation or transformation upon irradiation, potentially contributing to the observed spectral changes [40]. Furthermore, it is noticed that no new carbonyl or hydroxyl bands (typically observed around 1700–1750 cm^−1^ and 3200–3600 cm^−1^, respectively) were detected in any of the irradiated samples. This suggests that, within the dose range investigated (5–15 kGy), oxidation does not dominate the degradation mechanism, or that any oxidation products formed are below the detection limit of ATR-FTIR. This observation further suggests the predominance of chain scission and crosslinking as the primary competing mechanisms in this system. Other modifications are observed at the Si-O vibration peaks, which are associated with HNTs illustrated in Figure 4. The shifts may be interpreted as a result of improved dispersion or stronger interfacial interaction between HNTs and the ABS matrix upon irradiation. However, these shifts could also arise from minor structural rearrangements within the HNTs themselves (e.g., dealumination or changes in the octahedral sheet) or from local strain induced by the polymer matrix upon crosslinking, rather than from only improved interfacial bonding. This phenomenon is noticed in other irradiated polymer composites where fillers enhance interfacial bonding [41]. Indeed, studies on HDPE/rice husk ash composites show that EBI enhances the polymer–filler interface, which in turn influences the material’s overall performance [41]. Therefore, the spectral changes observed are consistent with structural rearrangements and oxidation processes as well as competing chain scission and crosslinking mechanisms, highlighting the critical role of HNTs in adjusting e-waste ABS composite response to EBI.

### 3.2. X-Ray Diffraction Analysis (XRD)

**Figure 5:**
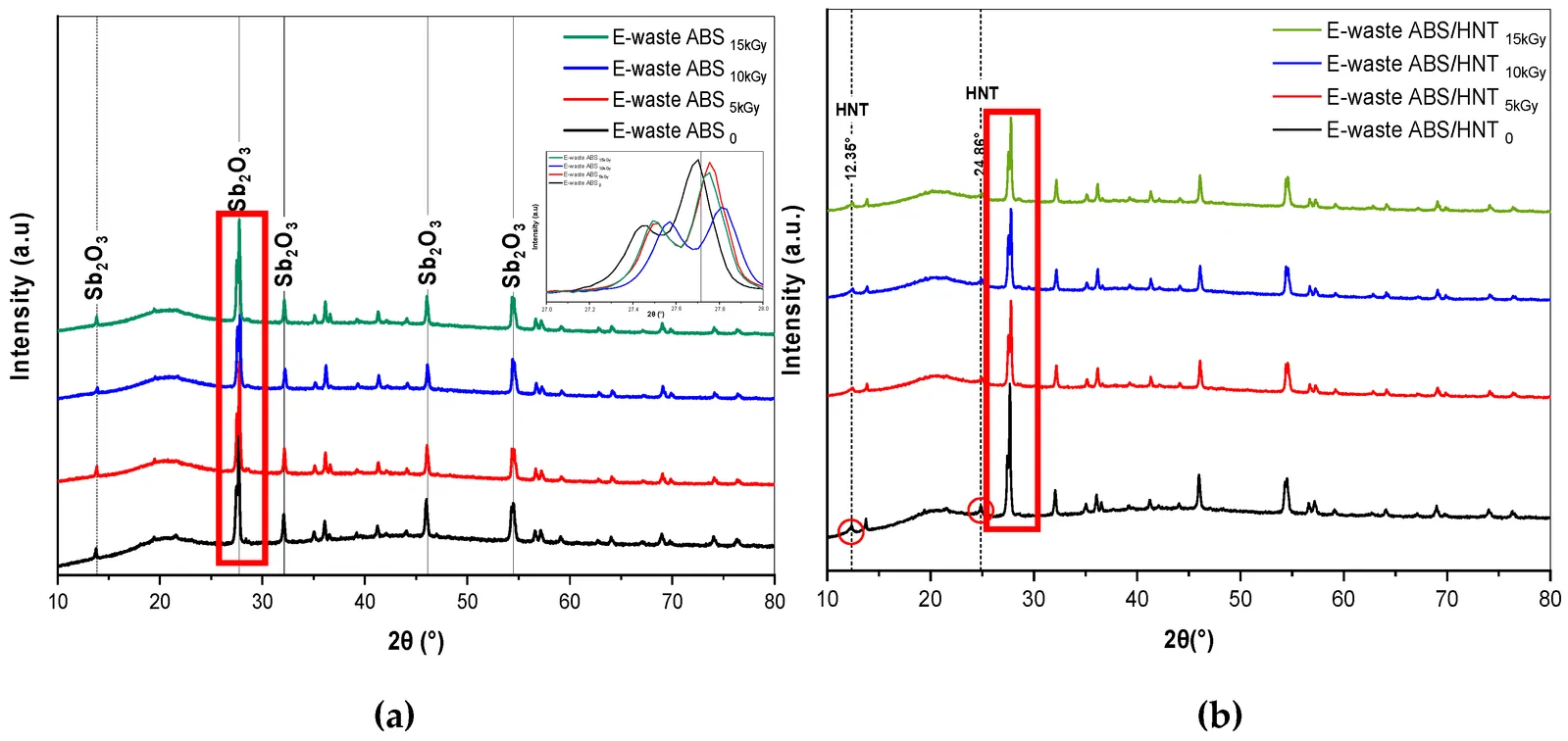
X-ray patterns of irradiated and unirradiated e-waste ABS (**a**) and e-waste ABS/HNTs (5 wt%) (**b**).

XRD patterns of both e-waste ABS and its HNT-reinforced composite shown in Figure 5a and Figure 5b, respectively, before and after EBI at 5, 10 and 15 kGy, display no shift in the position of the diffraction peaks over the investigated dose range given that ABS has an amorphous structure. Indeed, XRD results show that exposure to EBI at up to 15 kGy does not alter the position of the characteristic diffraction peaks of crystalline additives contained in the e-waste ABS, particularly at 2θ ≈ 27.70, 32.06, 46.00 and 54.48°, indicating the absence of irradiation-induced phase transformation. Nevertheless, a gradual decrease in peak intensity is observed as the irradiation dose increases, which may reflect changes in the structural order, crystallite size, or dispersion of these additives [42]. A comparable loss of diffraction intensity without peak shift has also been reported for inorganic fillers dispersed in irradiated polymer matrices, generally attributed to a reduction in crystallite size and to increasing disorder at the filler–matrix interface rather than to chemical alteration of the filler itself [42]. The potential chemical interaction between Sb_2_O_3_ and the brominated species did not proceed to the extent of producing a detectable crystalline SbOBr phase. While theoretically possible, this reaction is either kinetically hindered under our irradiation conditions or yields X-ray amorphous products that cannot be detected by conventional XRD.

After exposure to 5, 10, and 15 kGy, the characteristic diffraction peaks of Sb_2_O_3_ (especially the reflection at 2θ ≈ 27.70°) decreased in both materials. However, as shown in Table 3, this decrease was less noticeable in the e-waste ABS/HNTs composite than in the neat e-waste ABS. Normalized XRD patterns further show that, compared to the unfilled sample, the peak area at 2θ ≈ 27.70° in the composite dropped by about 18% at 5 and 15 kGy, while at 10 kGy the values were similar. These quantitative observations are consistent with a protective effect of HNTs on the crystalline structure of the additives from radiation-induced changes [43]. However, the use of XRD analysis alone cannot unambiguously prove such a protective mechanism, as the observed differences could also arise from variations in crystallite size, micro-strains, or preferred orientation induced by the presence of HNTs. Nevertheless, the consistently higher absolute peak areas in the composite (e.g., 6091 vs. 7423 a.u. at 15 kGy) support the interpretation that HNTs mitigate radiation-induced disorder in the Sb_2_O_3_ phase. This is further supported by the literature data showing that nanofillers such as clay, silica, and HNTs can reduce radiation-induced damage to co-dispersed crystalline additives through mechanisms including (i) dilution effects, (ii) preferential absorption of radiation energy by the filler, and (iii) restricted chain mobility in the interphase region, which limits radical migration and subsequent crystalline degradation [43]. Future work using complementary techniques such as TEM or XPS would be valuable to confirm the underlying mechanism.

### 3.3. Thermogravimetric Analysis (TGA)

**Figure 6:**
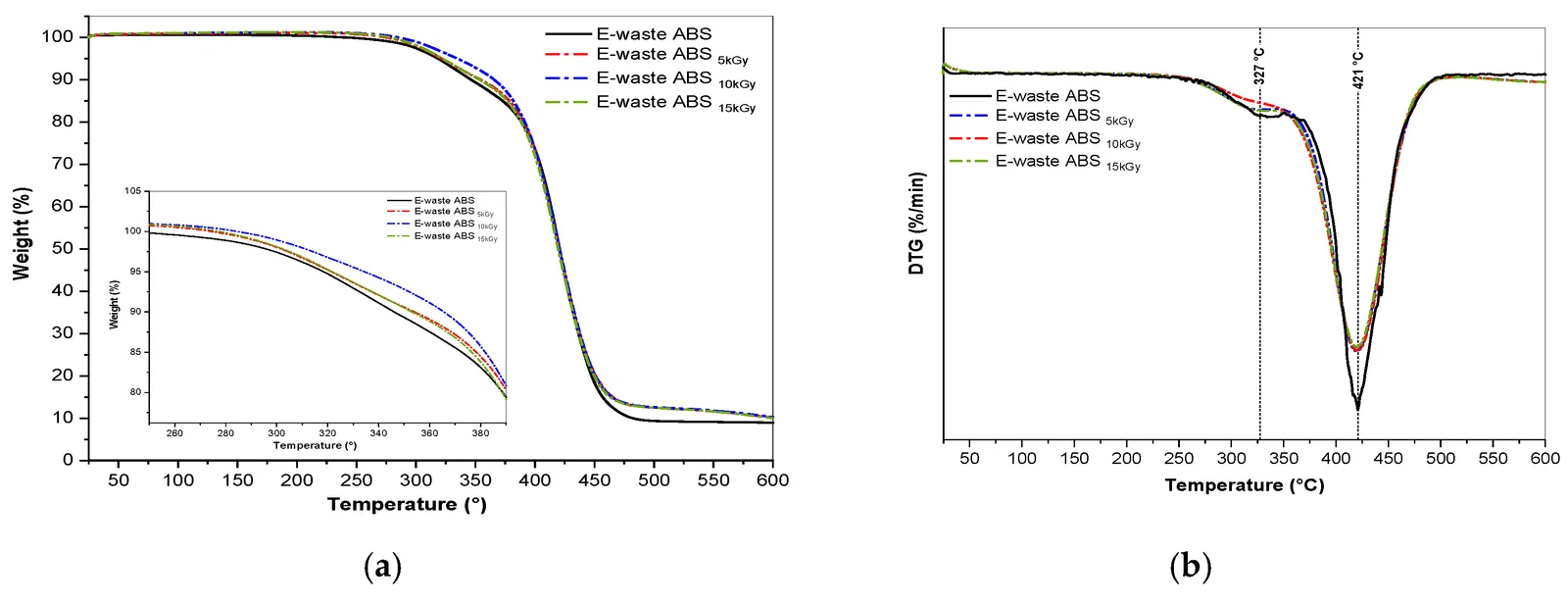
TGA: (**a**); DTG: (**b**) thermograms of e-waste ABS before and after EBI at 5, 10 and 15 kGy.

**Figure 7:**
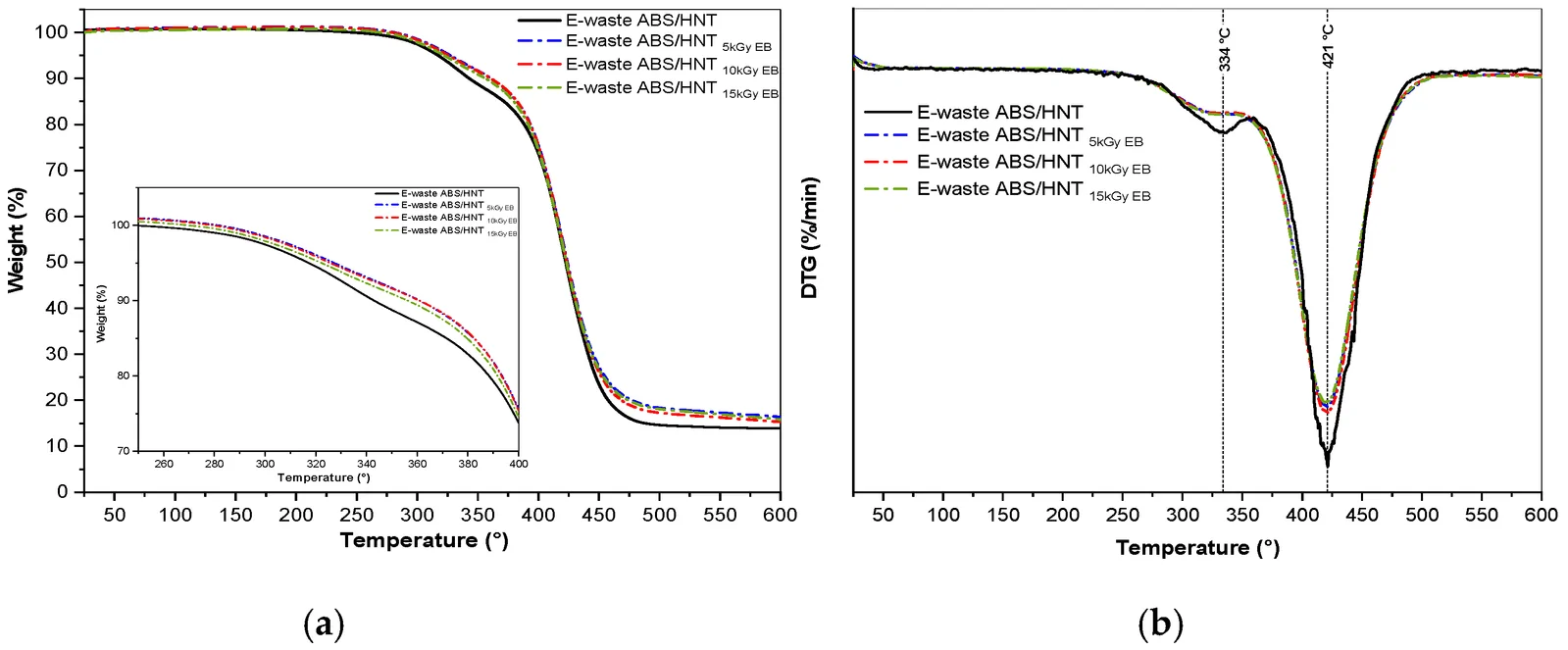
TGA: (**a**); DTG: (**b**) thermograms of e-waste ABS/HNTs (5 wt%) before and after EBI at 5, 10 and 15 kGy.

TGA/DTG analyses provide insight into the thermal degradation behavior of e-waste ABS and its HNT-reinforced composite before and after EBI at 5, 10, and 15 kGy. As illustrated in Figure 6a,b and Figure 7a,b, as well as in Table 4 summarizing the main data, T_5%_ (temperature at 5 wt% loss) of e-waste ABS displays the value of about 309 °C, which is much lower than the virgin resin (360–390 °C) [23]. This significant decrease in T_5%_ representing the material thermal stability is due to thermal histories experienced during the materials service life and also to melt recycling, promoting polymer chain scission resulting in molecular weight decrease, besides the depletion of additives incorporated in the polymer matrix such as thermal and UV stabilizers, lubricants, pigments, and antistatic and nucleating agents [9]. An additional contributing factor is the early decomposition of the brominated flame-retardant compound, which generates active radicals in the gas phase at relatively low temperatures, prior to further decomposition of the ABS matrix, as part of its flame-inhibition mechanism acting in the vapor phase [44]. After exposure to EBI at 10 kGy, the value of T_5%_ increases by 25 °C passing from the initial value of 309 °C to almost 334 °C (Table 4). This result suggests the dominance of recombination reactions of the free radicals taking place in the polybutadiene phase, thus promoting crosslinking, which further restricts polymer chain mobility and delays the beginning of thermal degradation. However, it is important to note that chain scission in the polybutadiene phase can also generate low-molecular-weight fragments that may act as plasticizers, potentially lowering T_5%_ and T_g_. The observed increase in T_5%_ at 10 kGy therefore reflects the competition between these two opposing effects, with crosslinking prevailing at this dose. At 15 kGy, we observe only a very slight increase in T_5%_ by 3 °C compared to that of the unirradiated sample. It is reasonable to assume that chain scission and oxidative degradation begin to compete more significantly, a common occurrence in polymer radiation chemistry where there is a balance between crosslinking and degradation depending on the dose [9]. The potential plasticizing effect of butadiene degradation fragments may also contribute to this plateau, as the accumulation of low-molecular-weight species at higher doses could offset the thermal stabilization provided by crosslinking. However, at 5 kGy, the decrease in T_5%_ is more pronounced with a recorded value of 302 °C compared to the unirradiated one, clearly demonstrating the dominance of the chain splitting mechanism. In this case, the generation of short-chain fragments from polybutadiene scission may act as internal plasticizers, further reducing thermal stability. The quantity of residue tends to decrease slightly for the irradiated samples. This could be ascribed to an influence of the irradiation on the char structure. However, the quantity of residue remains significant. This is consistent with previous studies on WEEE-derived ABS, which reported high concentrations of bromine and antimony originating from flame-retardant additives [4]. During thermal degradation, these elements may interact to form various thermally stable antimony-halogen species and other inorganic compounds, thereby contributing to the increased residual mass [4]. Although no elemental analysis of the residue was performed in this study, we assumed that the contribution of brominated and antimony-containing compounds to the residual mass is therefore inferred from the literature and from the known composition of e-waste ABS, rather than directly measured.

Furthermore, it is important to consider the potential volatilization of antimony tribromide (SbBr_3_), which has a boiling point of approximately 288 °C. Given that T_5%_ occurs at around 300–330 °C in our systems, SbBr_3_ may partially volatilize during thermal degradation, contributing to mass loss and potentially explaining part of the observed decrease in residue for irradiated samples. This volatilization may compete with the formation of thermally stable antimony-halogen species, and the net residual mass reflects the balance between these two processes. The addition of HNTs leads to a modest but consistent improvement in thermal stability of e-waste ABS before irradiation by almost 10 °C, with the T_5%_ values ranging between 319 and 327 °C as shown in Table 4 and Figure 7a,b. This enhancement can be attributed to the barrier effect of HNTs, which limits heat transfer and volatile diffusion, and also to filler–matrix interactions [45]. The DTG curves presented in Figure 7a,b support the thermal stability improvements observed for the HNT-reinforced composite. Compared to the unfilled e-waste ABS, the composite exhibits two distinct changes: a slight shift in Tmdr (temperature at maximum degradation rate) toward higher temperatures, and a noticeable reduction in peak intensity. These features indicate that the presence of HNTs slows down the degradation process, allowing the composite to decompose in a more gradual and controlled manner. The shift to higher temperature suggests that the nanotubes act as a thermal barrier, delaying the onset of the most rapid decomposition stage, while the lower peak intensity reflects a reduced rate of mass loss during that stage. In addition, the composite shows a higher residual mass (15–17%) compared to the unfilled matrix. This may be due to the initial HNT content, as these thermally stable inorganic nanotubes lose only about 15 wt% of their mass at 600 °C and remain largely intact within the residue after the polymer has fully degraded [46]. This increased residual mass is consistent with the literature data and is expected, since HNTs are physically incorporated rather than chemically modified. This effect is essentially independent of the irradiation dose, which is reasonable because HNTs act primarily as a physical barrier and do not chemically interact with radiation-induced radicals; thus, their thermal stabilization mechanism remains unchanged across the dose range studied. This increased amount of residue is beneficial from a flame-retardancy point of view, as mineral fillers are known to promote the formation of protective char layers that slow down heat and mass transfer during thermal degradation [23]. The effect of EBI on the residual mass is limited. TGA/DTG results indicate that EBI induces dose-dependent modifications in the thermal degradation behavior of e-waste ABS systems, with intermediate doses promoting crosslinking and improved thermal stability, while higher doses promote competing degradation mechanisms [47]. Furthermore, the plasticizing effect of low-molecular-weight fragments generated by polybutadiene chain scission, discussed earlier in relation to T_5%_, is also consistent with the T_g_ trends observed by DSC, where a decrease in T_g_ at higher doses reflects the competition between crosslinking and chain scission. Combining HNT reinforcement with controlled EBI offers an effective approach to improve the thermal stability of e-waste ABS, supporting its recycling into higher-value materials.

### 3.4. Differential Scanning Calorimetry (DSC)

**Figure 8:**
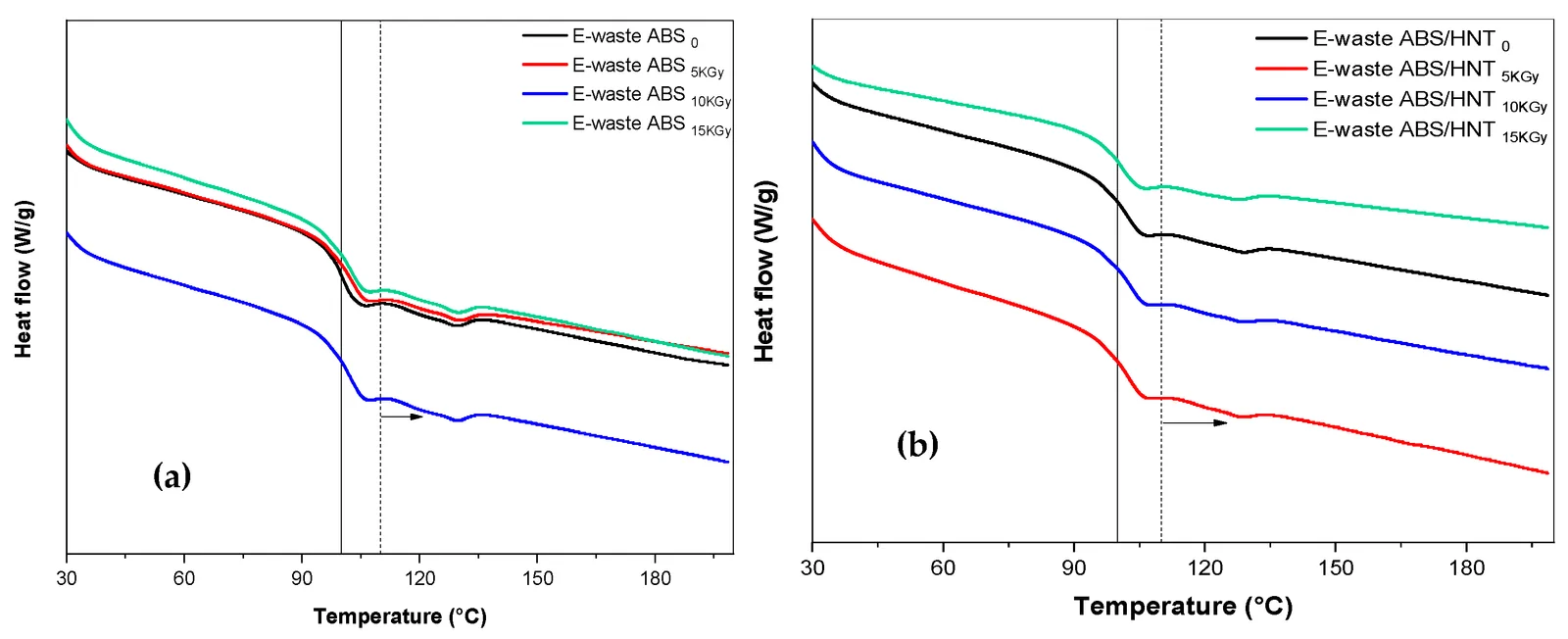
DSC thermograms of e-waste ABS (**a**) and e-waste ABS/HNTs (5 wt%) (**b**) recorded before and after irradiation at 5, 10 and 15 kGy.

In this study, we used DSC to determine the glass transition temperature (T_g_) of e-waste ABS and its HNT-reinforced composite, before and after EBI at 5, 10, and 15 kGy. Table 5 summarizes the T_g_ values of the samples with the dose whereas Figure 8a,b present the DSC thermograms of e-waste ABS and its composite, respectively, at various doses. The T_g_ values are indicated by dashed guide lines for ease of identification. From Table 5, T_g_ of e-waste ABS is initially about 109.8 °C, being almost the same value reported by de Oliveira et al. [48], which is nearly 110 °C. However, after exposure to EBI, three different effects emerged: the first one, observed at 5 kGy, is the significant decrease in the T_g_ value to roughly 102.8 °C. This decrease can be attributed to the chain scission mechanism induced by EBI, which further enhances chain mobility as reported by Mansouri et al. [49]. This decrease is also consistent with the TGA results presented earlier, where the generation of low-molecular-weight fragments from polybutadiene chain scission may act as internal plasticizers, reducing T_g_. The second one occurs at 10 kGy, where the T_g_ value slightly increases to 111.6 °C, which may indicate that crosslinking becomes the main effect, forming a network, thus restricting chain mobility. The last effect is noted at 15 kGy, where the T_g_ value is roughly 110.4 °C, close to that of the unirradiated sample, suggesting the occurrence of both phenomena of scission and recombination of the free radicals in the polymer matrix at the same reaction rate. As a result, the molecular mobility of chains is limited, thus inducing constant T_g_ values.

Adding HNTs to the e-waste ABS matrix may affect T_g_. Indeed, HNTs act as a physical barrier, restricting chain movement and raising T_g_ of the unirradiated composite sample by about 4 °C. This effect of nanofillers on thermal properties is well-documented [50]. However, the DSC data reported in Table 3 reveal that the irradiated composite samples behave as the unfilled one. Whatever the dose, T_g_ remains unchanged and presents almost the same value as the unirradiated composite one within the limits of standard deviation. From the above results, it clearly appears that the EBI of e-waste ABS initially breaks chains, then links them, while adding HNTs stabilizes the matrix, preventing the initial drop in T_g_ and making the composite material more resistant to changes from irradiation.

### 3.5. Tensile Tests

**Figure 9:**
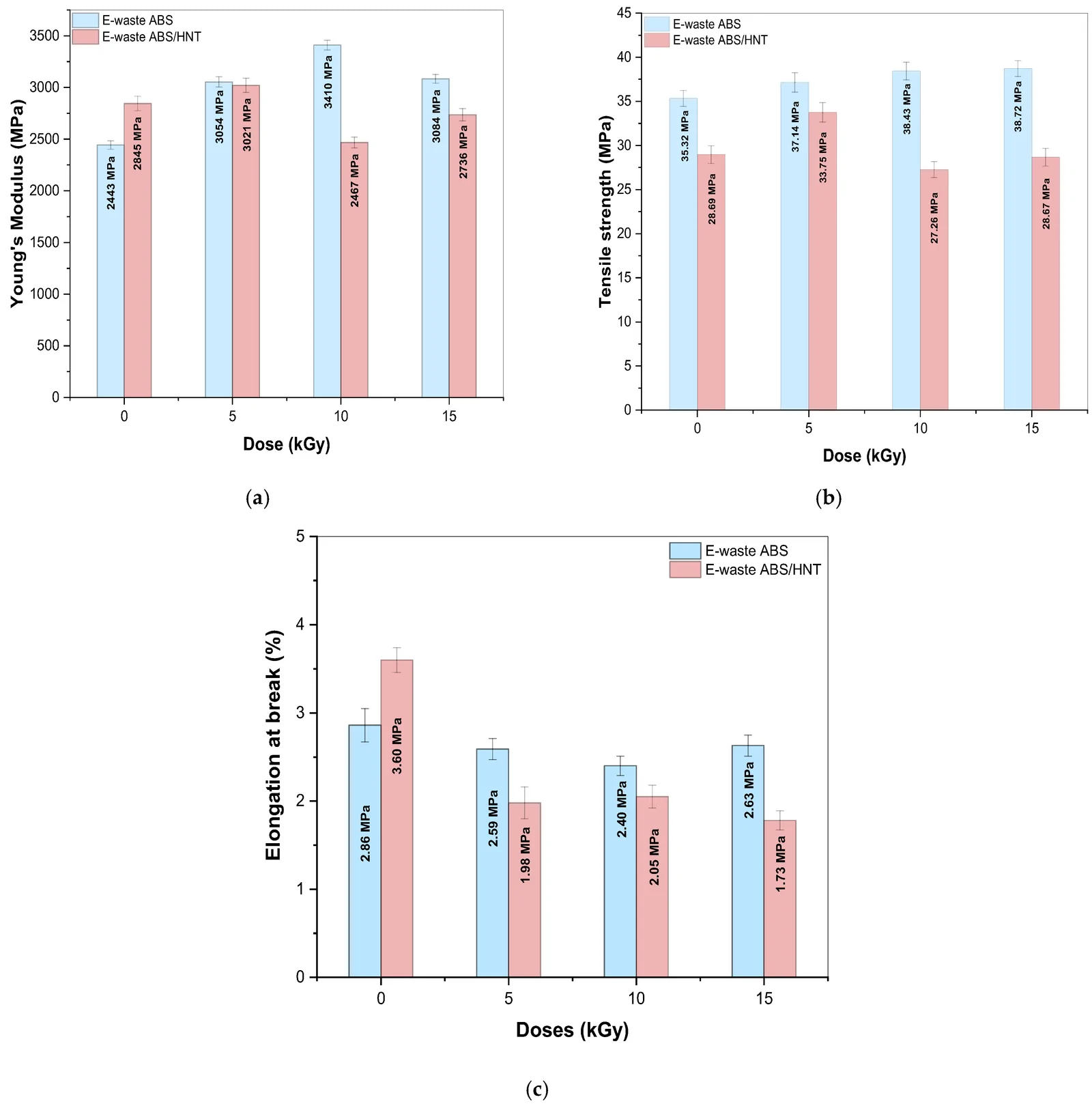
Tensile strength (**a**); Young’s modulus (**b**); and elongation at break (ε_b_) (**c**) of e-waste ABS and e-waste ABS/HNTs (5 wt%) before and after EBI at 5, 10 and 15 kGy.

Figure 9a–c displays the curves of tensile strength at yield (TS), Young’s modulus (E) and elongation at break (εb) of e-waste ABS and its HNT-reinforced composite, before and after EBI at 5, 10, and 15 kGy. The results reveal distinct responses to EBI for e-waste ABS versus HNT-reinforced composite, clearly indicating the complex relationships between radiation-induced chemistry and the presence of clay nanofillers. To provide a more complete picture of the mechanical behavior relevant to structural applications, we also examine the TS/E ratio (i.e., the strength-to-stiffness ratio), which reflects the material’s ability to sustain load relative to its rigidity. The corresponding results are presented in Table 6. A higher TS/E ratio indicates a more ductile-like response, where the material can withstand stress without excessive stiffness, whereas a lower ratio suggests a stiffer, more brittle behavior. This ratio is particularly useful for comparing materials where both strength and stiffness must be balanced for load-bearing applications. Although our discussion focuses on qualitative trends in mechanical behavior, to clearly show data variability, average values with standard deviation bars are added to all figures (Figure 9a–c). Standard deviation values are also included in Table 6 for the TS/E ratios.

For e-waste ABS, a slight enhancement in tensile properties is observed upon irradiation, with an optimal dose identified at 10 kGy. From Figure 9a, it is observed that TS increases from approximately 35 MPa for the unirradiated sample to nearly 38 MPa at 10 kGy. Concomitantly, E increases from nearly 2443 MPa, to over 3410 MPa (Figure 9b). This significant increase is due to radiation-induced crosslinking of the polymer chains. At 10 kGy, EBI promotes the formation of covalent bonds between adjacent polymer chains, creating a three-dimensional network. This network enhances the material’s resistance to deformation under load and subsequently increases both its strength (TS) and stiffness (E). Consequently, the TS/E ratio for e-waste ABS decreases from approximately 14.4 × 10^−3^ (unirradiated) to 11.2 × 10^−3^ at 10 kGy (Table 6), indicating that the material becomes relatively stiffer and less ductile at the optimal dose, as expected from a crosslinked network. However, at 15 kGy, a slight decline in both TS and E is observed. The decline can be explained by initiation of the chain scission mechanism, which competes with and, at a certain threshold, eliminates the beneficial effects of crosslinking. At 15 kGy, the TS/E ratio recovers slightly to approximately 12.5 × 10^−3^, reflecting the partial restoration of chain mobility due to competing chain scission. This competition between crosslinking and chain scission is a characteristic feature of polymer irradiation, where an optimal dose exists for property enhancement [51].

The addition of HNTs to e-waste ABS modifies the trend observed for the unfilled material. The HNT-reinforced composite exhibits lower TS and E values than e-waste ABS, and its optimal irradiation dose shifts to a lower value of 5 kGy. Regarding the TS/E ratio for the composite, the unirradiated e-waste ABS/HNTs sample shows a value of approximately 10.1 × 10^−3^, which is lower than that of the unfilled polymer (14.4 × 10^−3^). This indicates that the addition of HNTs increases stiffness more significantly than strength, consistent with the reinforcing effect of rigid mineral fillers. At 5 kGy, where the composite exhibits its optimal mechanical response, the TS/E ratio increases to approximately 11.2 × 10^−3^, suggesting that low-dose irradiation partially restores ductility while maintaining strength. At higher doses (10 and 15 kGy), the TS/E ratio decreases again, reflecting the dominance of interfacial degradation and embrittlement. These trends are summarized in Table 6. From a structural application perspective, the TS/E ratio highlights a trade-off: while the unfilled e-waste ABS at 10 kGy achieves the highest absolute strength and stiffness, its reduced TS/E ratio indicates a more brittle behavior. In contrast, the HNT-reinforced composite at 5 kGy offers a better balance between strength and ductility (higher TS/E), which may be preferable for applications requiring impact resistance or energy absorption. This behavior can be explained by several factors which are related to the processing of the composite material and the radiation chemistry in the presence of mineral nanofillers [21,52].

Indeed, the addition of 5 wt.% HNTs to e-waste ABS significantly modifies both the mechanical performance and the radiation response of the composite. This results in lower values of tensile strength and Young’s modulus compared with the unfilled e-waste ABS. Based on the literature on polymer–nanofiller composites, a possible explanation is the development of local stress concentrations at the filler–matrix interface, particularly in regions where filler dispersion may be less homogeneous [21,52,53]. Such interfacial regions can act as preferential sites for damage initiation under tensile loading and consequently contribute to a reduction in tensile strength. Similarly, possible local HNT clustering may increase the severity of these stress concentrations. However, these morphological features remain a hypothesis in the present work and cannot be confirmed without direct microscopic observations. The decrease in elongation at break is consistent with the increased rigidity and reduced ability of the composite to undergo plastic deformation before failure, which is commonly observed following the incorporation of mineral fillers into polymer matrices. Concerning the radiation behavior, an interesting shift is observed. While the optimal irradiation dose for unfilled e-waste ABS is 10 kGy, the composite shows its best overall mechanical response at a lower dose of 5 kGy. This suggests that the presence of HNTs modifies the radiation response of the system. According to the literature [54], interfacial regions between mineral fillers and polymer matrices can influence radiation-induced processes. However, the specific mechanism responsible for the lower optimal dose observed in the present HNTs/ABS system cannot be conclusively established from the available characterization techniques. The observed dose dependence may result from the combined effects of radiation-induced chain scission, crosslinking, and polymer–filler interfacial interactions. Therefore, the proposed interfacial contribution should be considered as a possible interpretation rather than a directly demonstrated mechanism.

### 3.6. Rheological Measurements

**Figure 10:**
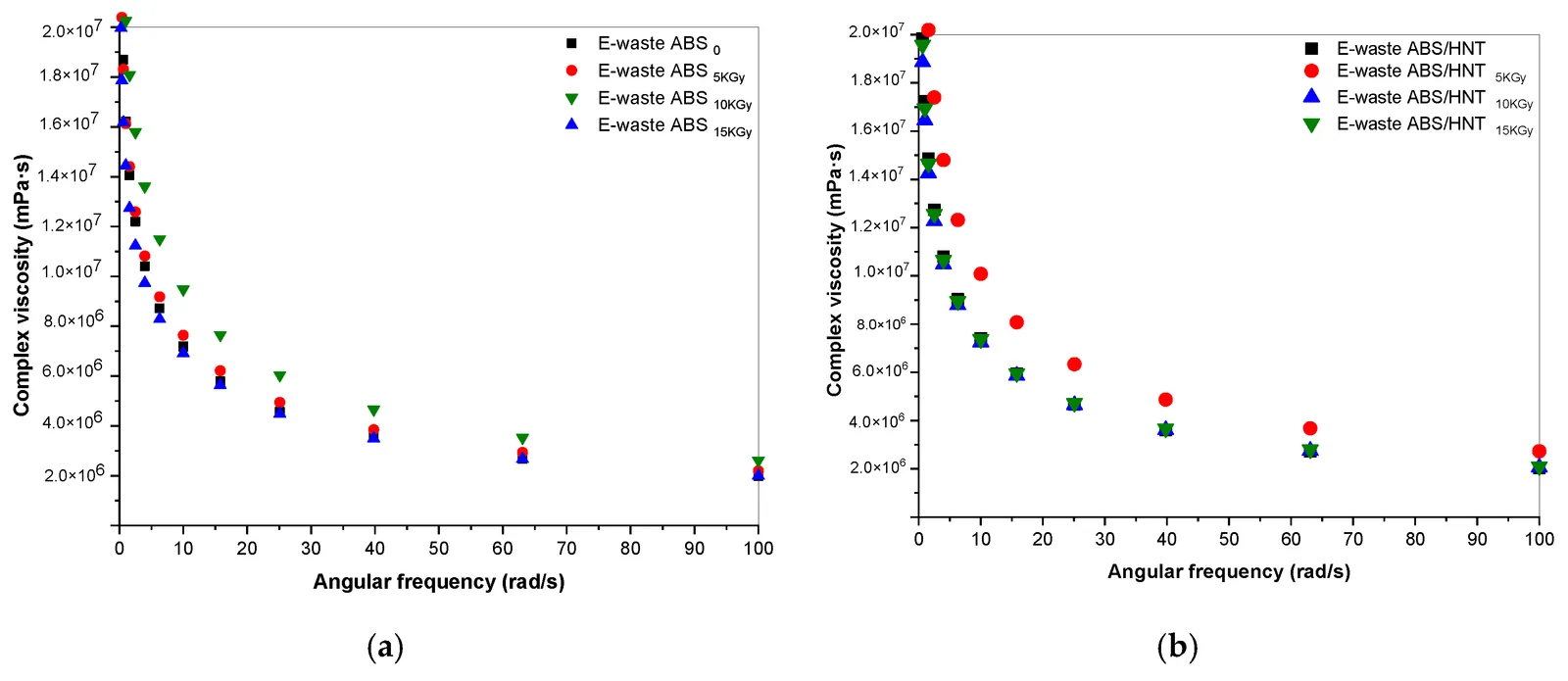
Complex viscosity of e-waste ABS (**a**) and e-waste ABS/HNTs (wt.%) (**b**) before and after EBI at 5, 10 and 15 kGy.

The effect of EBI on the complex viscosity (η*) of e-waste ABS and e-waste ABS/HNTs at various doses, 5, 10 and 15 kGy, is clearly illustrated in Figure 10a and Figure 10b, respectively. In both cases, it decreases as the frequency increases, indicating a shear-thinning behavior, which is a common behavior of polymer melts. Figure 10a shows the variation in η* of e-waste ABS with dose. It is observed that the sample irradiated at 10 kGy maintains a higher overall complex viscosity curve across the entire frequency range compared to the unirradiated sample and to those irradiated at 5 and 15 kGy. This indicates that, rather than a simple reduction, the 10 kGy sample exhibits a net increase in viscosity relative to the other doses. This deviation can be attributed to a shift in the dominant radiation-induced mechanism. At 10 kGy, the dose approaches a threshold where cross-linking begins to compete with chain scission, leading to the formation of a more interconnected molecular network that mitigates the viscosity drop. In contrast, the decrease in viscosity at 5 and 15 kGy is primarily governed by chain scission, which reduces molecular weight and results in a lower overall complex viscosity curve. The fact that the 15 kGy sample shows a viscosity drop comparable to that at 5 kGy suggests that, beyond the 10 kGy threshold, chain scission again becomes the dominant mechanism, likely due to the onset of main-chain degradation at higher doses. From these findings, chain scission and cross-linking occur simultaneously, and the balance between these competing processes is strongly dose-dependent [55,56,57]. Furthermore, a comparison of Figure 10a,b reveals that the decrease in complex viscosity with increasing irradiation dose is significantly more pronounced for the unfilled e-waste ABS than for the HNT-reinforced composite. For instance, at an irradiation dose of 5 kGy, the e-waste ABS/HNTs composite exhibits a markedly higher complex viscosity (approximately 100 × 10^5^ mPa·s) recorded at 10 rad·s^−1^ compared to the unfilled e-waste ABS (76 × 10^5^ mPa·s). To prove this stabilizing effect, the complex viscosity data were fitted to the Carreau–Yasuda model, and the extracted parameters (η_0_ and λ) confirm that the HNT-filled sample at 5 kGy exhibits a significantly higher zero-shear viscosity (76.11 Pa·s) and longer relaxation time (0.0613 s) compared to the unfilled system (η_0_ = 38.65 Pa.s and λ = 0.0455 s), indicating a more robust network structure. This suggests that the presence of HNTs promotes the formation of a physical or chemical network within the polymer matrix, even at relatively low irradiation doses. Such behavior is likely attributed to interfacial interactions between the HNTs and the ABS matrix [56], which may restrict polymer chain mobility and enhance structural integrity, thereby mitigating the irradiation-induced degradation observed in the unfilled system. Similarly, in Figure 10b, the sample irradiated at 5 kGy maintains a distinctly higher complex viscosity curve across the entire frequency range compared to the other doses, indicating that the incorporation of HNTs modifies the polymer’s response to EBI by enhancing the crosslinking efficiency at this dose. Figure 10b further reveals that the irradiated sample at 5 kGy exhibits a notably slower viscosity reduction compared to the other doses. This behavior suggests that the incorporation of HNTs modifies the polymer’s response to EBI. It should be noted that the radiation-induced changes (chain scission and cross-linking) are permanent chemical modifications; however, the physical polymer–filler interactions are reversible upon heating. This is consistent with studies showing that HNTs can act as radiation stabilizers, by absorbing energy at the polymer–filler interface. The role of such nanofillers, in protecting the matrix, highlights the complex dose-dependent nature of radiation effects in polymer composites [21].

**Figure 11:**
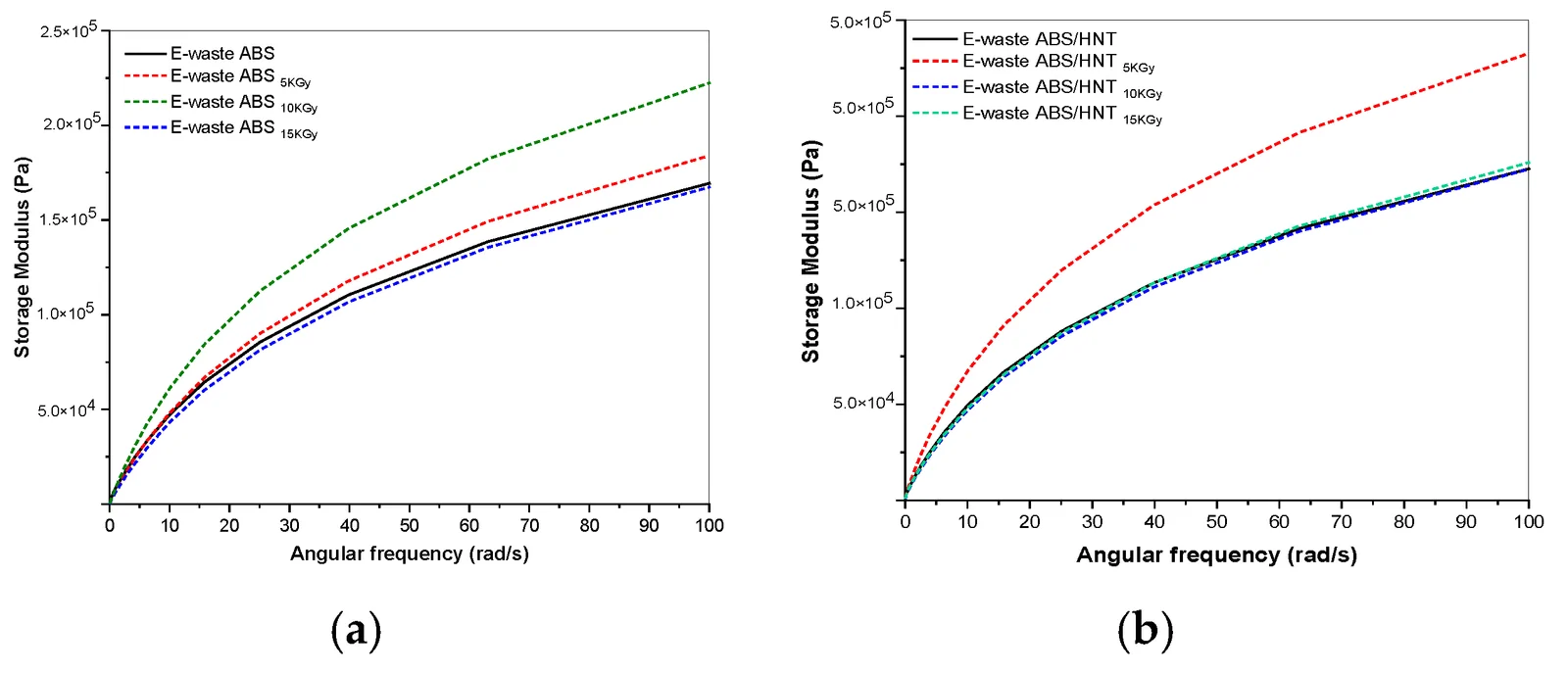
Storage modulus of e-waste ABS (**a**) and e-waste ABS/HNTs (**b**) before and after EBI at 5, 10 and 15 kGy.

Figure 11a,b shows the effect of EBI on the storage modulus (G’) of e-waste ABS and its HNT-reinforced composite, respectively. The rheological analysis shows that G’ increases regularly with frequency for all samples, a characteristic behavior of polymer melts in the terminal flow region. However, a distinct radiation-dose dependence is observed that reveals the underlying microstructural changes. For the unfilled e-waste ABS, the irradiated sample at 10 kGy exhibits the highest G’ values across the measured frequency range, followed by the sample irradiated at 5 kGy. In contrast, the G’ curve for the unirradiated sample and the irradiated one at 15 kGy are almost identical and significantly lower than those at 10 kGy and, to a lesser extent, at 5 kGy. This trend is consistent with the viscosity data, where the 10 kGy sample exhibits a slower decrease in viscosity. A higher G’ indicates a more elastic, solid-like network. This is strong evidence that at 10 kGy, cross-linking becomes the dominant effect, creating a stronger elastic network that resists flow. The intermediate effect at 5 kGy suggests the onset of this process. For instance, studies on ABS reported that while low to moderate irradiation doses can promote crosslinking and improve mechanical properties, excessively high doses (e.g., ≥200 kGy) lead to a significant reduction in properties due to chain scission [32].

The near-identical G’ values at 0 and 15 kGy for e-waste ABS suggest that by 15 kGy, the degradative effects of chain scission start to counterbalance the crosslinking, returning the network density to a state similar to the unirradiated material. For the HNT-reinforced composite, a different trend emerges. The sample irradiated at 5 kGy demonstrates the most elevated G’ curve, while the curves for the samples irradiated at 10 and 15 kGy, as well as the unirradiated sample, are closely grouped and almost superimposed. This finding provides a crucial insight into the previous viscosity result for the composite. HNT nanoparticles appear to act as multi-functional sites. At the optimal dose of 5 kGy, radiation likely promotes highly efficient polymer–filler crosslinking dramatically enhancing the elastic polymer–filler network and resulting in the highest G’. The concept of using controlled irradiation to tune the properties of polymer nanocomposites is a well-established strategy. As noted in recent reviews, the simultaneous effect of nanofillers and controlled electron beam irradiation can synergistically enhance the mechanical and rheological properties of polymer systems by improving the interfacial adhesion [58]. At higher doses (10, 15 kGy), degradation by chain scission may begin to counterbalance or damage this optimized network, returning the properties to a baseline similar to the unirradiated composite. This is analogous to observations in other irradiated polymer systems where an optimal dose exists for property enhancement, beyond which over-irradiation leads to degradation [59]. Thus, rheology provides crucial insight into the melt-state network formation driven by irradiation and filler presence. It corroborates the tensile findings by identifying 5 kGy as the optimal dose for the HNT-reinforced composite. The results indicate that while for e-waste ABS the crosslinking effect becomes rheologically dominant at 10 kGy, the presence of HNTs shifts this optimum to a lower dose of 5 kGy, highlighting the favorable action of the nanofiller in promoting network formation at lower radiation doses.

## 4. Conclusions

This study investigated the combined effects of low-dose electron beam irradiation (EBI) and halloysite nanotubes (HNTs) on the properties of recycled ABS recovered from electrical and electronic waste (e-waste). The results demonstrate that EBI at 10 kGy enhanced the tensile strength and Young’s modulus of unfilled e-waste ABS by approximately 9% and 40%, respectively, and increased thermal stability (T_5%_) by about 25 °C, consistent with radiation-induced crosslinking. The incorporation of 5 wt.% HNTs shifted the optimal irradiation dose to 5 kGy, at which the composite exhibited the highest storage modulus and improved thermal stability relative to unirradiated samples. These findings suggest a potential route for valorizing e-waste ABS through a combination of nanofiller reinforcement and controlled irradiation. Several limitations affect the practical applicability of this approach. First, the mechanistic interpretation of HNTs’ effects on the radiation response remains partly hypothetical. Complementary techniques such as electron spin resonance (ESR), transmission electron microscopy (TEM), or X-ray photoelectron spectroscopy (XPS) would be required to confirm the proposed interfacial and radical-mediated effects. Second, the study was conducted on a single homogenized batch of e-waste ABS sourced from a specific geographical region and comprising only white-colored components. The presence of brominated flame retardants, antimony trioxide, and other additives in this feedstock may significantly influence the radiation response, as suggested by the XRD results. Batch-to-batch variability was not assessed before industrial implementation. Third, the property improvements are modest in absolute terms and are accompanied by trade-offs. While irradiation at optimal doses improved stiffness and thermal stability, it also reduced elongation at break and increased brittleness, as reflected in the decreased TS/E ratio. For the HNT-reinforced composite, tensile strength was slightly lower than that of the unfilled matrix, likely due to filler aggregation and stress concentration effects, although direct microstructural confirmation was not performed. Moreover, the influence of irradiation on flame-retardant performance, which is critical for electrical and electronic applications, was not evaluated. Given that e-waste ABS typically contains brominated flame retardants, any upcycling strategy must verify that flammability characteristics are not adversely affected. Finally, all experiments were conducted at laboratory scale using compression-molded films. Moreover, the long-term stability of the irradiated materials, including potential post-irradiation aging effects, was not assessed. In view of these limitations, the proposed approach should be regarded as a promising but not yet fully validated upcycling strategy. The combination of low-dose EBI and HNTs offers a solvent-free, additive-free method to partially restore the mechanical and thermal performance of recycled e-waste ABS, supporting circular economy objectives.

## Figures and Tables

**Table 1:** Elemental chemical composition of e-waste ABS determined by EDX analysis [23].

Elemental Chemical Composition	Carbon (C)	Bromine (Br)	Antimony (Sb)	Titanium (Ti)	Chlorine (Cl)	Silicon (Si)
Composition (%)	87	8.5	2.8	0.8	0.7	0.2

**Table 2:** Variation in band ratios (I_3026/1602_, I_2919/1602_, and I_2850/1602_) with irradiation dose for e-waste ABS and e-waste ABS/HNTs composite.

	E-Waste ABS			E-Waste ABS/HNTs		
Dose (kGy)	I_3026_/I_1602_	I_2919_/I_1602_	I_2850_/I_1602_	I_3026_/I_1602_	I_2919_/I_1602_	I_2850_/I_1602_
0	1.003	0.897	0.976	1.002	0.741	0.938
5	1.001	0.883	0.969	1.014	0.794	0.999
10	1.018	0.926	0.993	1.010	0.769	0.950
15	1.005	0.884	0.974	1.023	0.774	0.960

**Table 3:** Comparative data of peak area at 27.70° for e-waste ABS and e-waste ABS/HNT composite.

	Diffraction Peak Area of Sb_2_O_3_ at 2θ = 27.70° (a.u.)	
Doses (kGy)	E-Waste ABS	E-Waste ABS/HNT (5 wt%)
0	8325	9000
5	7603	6237 (~−18%)
10	6312	6213 (~−1.6%)
15	7423	6091 (~−17.9%)

**Table 4:** Values of T_5%_ and residue (%) at 600 °C of e-waste ABS and e-waste ABS/HNTs (5 wt%) before and after e-beam irradiation at 5, 10 and 15 kGy.

Doses	0		5 kGy		10 kGy		15kGy	
	T_5%_ (°C)	% Residue	T_5%_ (°C)	% Residue	T_5%_ (°C)	% Residue	T_5%_ (°C)	% Residue
E-Waste ABS	309 ± 1.6	11.6 ± 0.1	302 ± 2.3	10.1 ± 0.9	334 ±1.8	10.3 ± 0.7	312 ± 2	10.2 ± 1.9
E-Waste ABS/HNTs (5 wt%)	319 ± 2.1	16.9 ± 1.3	327 ± 2.1	16.4 ± 0.5	326 ± 0.4	15.3 ± 1.9	323 ± 1.1	15.8 ± 0.8

**Table 5:** T_g_ values of e-waste ABS and e-waste ABS/HNTs (5 wt%) before and after EBI at 5, 10 and 15 kGy.

Doses	T_g_ (°C)			
	0	5 kGy	10 kGy	15 kGy
E-waste ABS	109.8 ± 1.1	102.8 ±1.7	111.6 ±1.4	110.4 ±1.1
E-waste ABS/HNTs (5 wt%)	113.2 ± 1.3	115.1 ±1.3	113.15 ±1.9	113.8 ±1.4

**Table 6:** Tensile strength/Young’s modulus ratio (TS/E) for e-waste ABS and e-waste ABS/HNTs at various irradiation doses.

Dose (kGy)	E-Waste ABS (TS/E)	E-Waste ABS/HNT (TS/E)
0	0.01446 ± 0.00122	0.01008 ± 0.00435
5	0.01216 ± 0.00275	0.01117 ± 0.00154
10	0.01127 ± 0.00197	0.01105 ± 0.00082
15	0.01256 ± 0.00543	0.01048 ± 0.00132

## Data Availability

The original contributions presented in this study are included in the article. Further inquiries can be directed to the corresponding author.

## Abbreviations

The following abbreviations are used in this manuscript:

EBI	electron beam irradiation
HNTs	Halloysite nanotubes
